# A plasma proteomic approach in patients with heart failure after acute myocardial infarction: insights into the pathogenesis and progression of the disease

**DOI:** 10.3389/fcvm.2023.1153625

**Published:** 2023-05-17

**Authors:** Yan Liu, Da Huang, Zhile Li, LiuFang Zhou, Tuan Cen, Baomin Wei, Liuqing Wei, Hongying Wu, Liye Su, Suren R. Sooranna, Xinshou Pan, ZhaoHe Huang

**Affiliations:** ^1^ Department of Cardiology, Affiliated Hospital of Youjiang Medical University for Nationalities, Baise, China; ^2^ Graduate School, Youjiang Medical University for Nationalities, Baise, China; ^3^ Department of Surgery and Cancer, Imperial College London, Chelsea and Westminster Hospital, London, United Kingdom; ^4^ Life Science and Clinical Research Center, Youjiang Medical University for Nationalities, Baise, China; ^5^ Affiliated Southwest Hospital, Youjiang Medical University for Nationalities, Baise, China

**Keywords:** acute myocardial infarction, plasma proteomics, heart failure, disease progression, biomarkers

## Abstract

**Aims:**

The pathogenesis of disease progression targets for patients with heart failure after acute myocardial infarction was investigated by using plasma proteomics.

**Methods:**

The plasma proteomes of acute myocardial infarction patients with (MI-HF) and without (MI-WHF) heart failure were compared. Each group consisted of 10 patients who were matched for age and sex. The peptides were analyzed by 2-dimensional liquid chromatography coupled to tandem mass spectrometry in a high definition mode. Parallel reaction monitoring (PRM) verified the selected target proteins.

**Results:**

We identified and quantified 2,589 and 2,222 proteins, respectively, and found 117 differentially expressed proteins (DEPs) (≥1.5-fold), when the MI-HF and MI-WHF groups were compared. Of these 51 and 66 were significantly up-regulated and down-regulated, respectively. The significant DEPs was subjected to protein–protein interaction network analysis which revealed a central role of the NF-κB signaling pathway in the MI-HF patients. PRM verified that MB, DIAPH1, VNN1, GOT2, SLC4A1, CRP, CKM, SOD3, F7, DLD, PGAM2, GOT1, UBA7 and HYOU1 were 14 proteins which were highly expressed in MI-HF patients.

**Conclusions:**

These findings showed a group of proteins related to the NF-κB signaling pathway in the pathogenesis of patients with poor outcomes after experiencing MI-HF. These proteins may be useful candidate markers for the diagnosis of MI-HF as well as help to elucidate the pathophysiology of this major cause of mortality in older patients.

## Introduction

Heart failure (HF) is associated with diverse etiologies and it has a complex clinical course (1). Hypertension and coronary artery disease are two of the most common causes of HF (2), especially in acute myocardial infarction (AMI). However, although the treatment for patients with MI has improved substantially in recent years, the incidence of HF after MI remains high, despite improvements in the survival rates (3–6). In this way, accurate and early identification of the etiology of cardiomyopathy could improve the outcome of HF patients (7). Therefore, the search for early biological markers of HF in the commonly sampled body fluids of patients is an important goal. Use of proteomics to identify potentially important proteins has previously proven to be a successful strategy for the early diagnosis of cancer (8).

22 of the most abundant proteins in human plasma account for around 99% of its total proteins, with a dynamic range of between 45 mg/ml for serum albumin and 1–10 pmol/ml for cytokines (9). Therefore, identification of candidate biomarkers within the 1% of the low abundance proteins that are related to pathogenesis for clinical abnormalities is a challenging endeavor (10–14). Network analysis is increasingly becoming a fruitful method of analysis in bioinformatics (15), and it can reveal the functional cross-links between genes associated with inflammation as well as diseases (16). In addition, network analysis has been able to identify potential targets that are important for understanding the mechanisms of certain diseases (17).

We used network analysis to compare the plasma proteome of AMI patients who had and did not have HF in order to provide insights into the pathogenesis of disease progression of this condition. This study aimed to search for potential biomarkers of HF after AMI as well as determining the possible mechanisms involved by investigating the plasma proteome.

## Materials and methods

### Study subjects

This study was approved by the Ethics Committee of the Affiliated Hospital of Youjiang Medical University for Nationalities, Baise, PR China, in accordance with the Helsinki Declaration. All the patients needed to sign an informed consent and this met criteria set out by the Helsinki Declaration. We enrolled 20 subjects aged 39–87 years who were hospitalized in the Cardiovascular Department of the Affiliated Hospital of Youjiang Medical University for Nationalities from 1 January, 2020 until 31 December, 2021.

The subjects were subdivided into two groups and their peripheral blood samples were used in this study. Ten patients were classed as the AMI with HF (MI-HF) group and 10 were enrolled in AMI without HF (MI-WHF) group and their blood samples were collected within 12 h first of diagnosis. Patients included in the AMI-HF group followed the latest guidelines for the diagnosis of AMI issued by the European Society of Cardiology in 2017 (18) and the 2021 ESC Guidelines for the diagnosis and treatment of acute and chronic HF (19). The patients excluded were those with rheumatic and congenital heart disease as well as those with dilated cardiomyopathy. The patients undergoing intravenous thrombolysis, having either systemic or local severe infection, auto-immunologic or blood system disease as well as those with severe kidney or liver disease or malignant disease were also excluded. Others with coronary stenting and coronary artery bypass grafting were not included.

The clinical data collected included age, gender, body mass index (BMI), smoking (smokers were defined as those who smoked at least one cigarette per day for more than one year) and drinking status (drinkers were defined as patients who consumed at least one alcoholic drink a day for a minimal period of six months). Data relating to hypertension, diabetes, total cholesterol (TC), triglyceride (TG), high density lipoprotein cholesterol (HDL-C), low-density lipoprotein cholesterol (LDL-C), extremely low-density lipoprotein cholesterol (VLDL-C), apolipoprotein A1, apolipoprotein B, lipoprotein a, homocysteine, urea, creatinine, uric acid, *β*2-microglobulin, cystatin C, creatinine clearance (CCr) and hs-CRP were also obtained from participants.

### Plasma sample collection and storage

A blood sample was obtained from each patient who were laid in the supine within 30 min after admission to the hospital. Samples were also obtained a week after their cardiac color ultrasound review that indicated EF < 40%. The samples were collected in 10 ml EDTA vacutainer tubes and these were inverted eight times and placed immediately on ice. The Plasma was separated by centrifugation at 1,500 g for 10 min at 4°C. Aliquots of these were stored at −80°C until further analysis.

## Experimental procedures

### Protein extraction

The plasma samples were thawed and the cellular debris were removed by centrifugation at 12,000 g at 4°C for 10 min. The supernatants were transferred into new centrifuge tubes. The top 14 proteins with highest abundance were removed by using Pierce™ Top 14 Abundant Protein Depletion Spin Column Kits (ThermoFisher Scientific, Shanghai, China). A BCA kit was used to measure the protein concentration by following the manufacturer's instructions.

### Trypsin digestion

The protein solution was reduced with 5 mM dithiothreitol for 30 min at 56°C and alkylated with 11 mM iodoacetamide for 15 min at room temperature in darkness prior to digestion. This solution was diluted by adding 100 mM triethylammonium bicarbonate to obtain a urea concentration of less than 2 M. Finally, trypsin was added to obtain a trypsin-to-protein mass ratio of 1:50 for the first digestion overnight. A second digestion with a trypsin-to-protein mass ratio of 1:100 was carried out for 4 h. Finally, the peptides were de-salted by using a C18 SPE column (Phenomenex, Torrance, USA).

### Liquid chromatography coupled to tandem mass spectrometry (LC-MS/MS) analysis

The tryptic peptides were dissolved in solvent A and loaded onto a home-made reversed-phase analytical column (25 cm length, 75 µm). The peptides were separated using a gradient from 5 to 25% of solvent B over 60 min, followed by 25 to 35% over 22 min which was increased to 80% for 4 min. It was then held at 80% for the last 4 min on an EASY-nLC 1200 UPLC system (Thermo Fisher Scientific). Solvents A and B were composed of 0.1% formic acid and 2% acetonitrile/ in water and 0.1% formic acid in 90% acetonitrile, respectively. The flow rate was kept constant at 450 nl/min. The separated peptides were analyzed in a Q Exactive^™^ HF-X mass spectrometer (Thermo Fisher Scientific) with a nano-electrospray ion source attachment. An electrospray voltage of 2.0 kV was applied. The full MS scan resolution was set to 60,000 and the scan range used was 350–1,600 m/z. 20 of the most abundant precursors were then selected for further analysis by using MS with a 30 s dynamic exclusion. The fragments were obtained by using HCD fragmentation at a normalized collision energy (NCE) of 28% and they were detected by using an Orbitrap at a resolution of 30,000. The fixed first mass and the automatic gain control (AGC) target were set at 100 m/z and 1E5, respectively. An intensity threshold of 3.3E4 and a maximum injection time of 60 ms were used.

### Database search

The obtained MS data were processed using the MaxQuant search engine (v.1.6.15.0). The tandem mass spectrums were searched against the human SwissProt database (20,422 entries) concatenated with a reverse decoy database. Trypsin/P was the cleavage enzyme specified which allowed for up to 2 missing cleavages. 20 ppm was set as the mass tolerance for precursor ions during the first search and 5 ppm was set for the main search. The mass tolerance for fragment ions was set as 0.02 Da. The fixed modification was specified as Carbamidomethyl on Cys and the variable modifications were specified as acetylation on the protein N-terminals and oxidation on Met. A false discovery rate (FDR) adjusted to < 1% was. We used the Benjamini-Hochberg to perform the FDR correction. We used the adjusted *P*-value (Q value) as FDR-corrected *P*-value for each differentially expressed candidate protein.

## Bioinformatics methods

### GO annotation

Gene Ontology (GO) is a major bioinformatics initiative that allows unification of the representation of gene and gene product attributes across different species. We derived a GO annotation proteome by using the UniProt-GOA database (http://www.ebi.ac.uk/GOA/). The identified protein IDs were first converted to their UniProt IDs and then these were mapped to GO IDs. If an identified protein was not annotated in the UniProt-GOA database, the InterProScan software package was used for annotation of its GO function based on its protein sequence alignment. Then the proteins were then classified according to three categories: biological process, cellular component and molecular function.

### Domain annotation

A protein domain is a conserved part of a given protein sequence and structure that is capable of evolving and assuming different function. It also exists independently from the rest of a protein chain. The identified protein domain functional descriptions can be annotated by using InterProScan (a sequence analysis application) based on its protein sequence alignment method and the InterPro domain database. The online software, InterPro, is a database that can integrate the diverse information regarding protein families, domains and functional sites (http://www.ebi.ac.uk/interpro/).

### KEGG pathway annotation

The Kyoto Encyclopedia of Genes and Genomes (KEGG) database is used to connect the known information on molecular interaction networks, including pathways and complexes (the “Pathway” database). It also incorporates the information regarding genes and proteins generated by specific genome projects (including the gene database). In addition, information associated with biochemical compounds and reactions (including compounds and reaction databases) are also stored. This database was, therefore, used to annotate the protein pathways.

### Statistical analysis

After classification of the proteins by their GO annotations, KEGG pathway analysis and the use of the InterPro database, we used a two-tailed Fisher's exact test employed to test the enrichment of the differentially expressed protein (DEGs) against all the proteins identified. In each case, the corrected *P*-values <0.05 were considered significant for the data obtained.

### Enrichment-based clustering

DEG functional classification (such as: GO, Domain, Pathway and Complex) were subjected to further hierarchical cluster analysis (HCA) based on collating all the categories obtained after enrichment along with their *P*-values. These were then filtered for those categories which were enriched in at least one of the clusters and they had a *P*-value <0.05. The filtered *P*-value matrix was then transformed using the function, *x* = −log10 (*P*-value). Then all the x values were z-transformed according to their functional category. The z scores obtained were then clustered by one-way CA (using the Euclidean distance and average linkage clustering) by using the Genesis software package. The cluster membership then drawn as a heatmap by using the “heatmap.2” function from the “gplots” R software package.

### Protein-protein interaction network

All the DEG database accession and sequences were searched using the STRING database (v 11.0) for any protein-protein interactions. The proteins belonging to the searched datasets were selected. STRING defines a metric called “confidence score” which is used to define the interaction confidence of the selections made. Interactions that had a confidence score ≥0.7 which reflected a high confidence were subsequently selected. The interaction network form STRING was then visualized by using the R software package, “networkD3”.

### PRM validation of proteomics

This project used the MS-based targeted proteome quantification technology that allowed parallel reaction monitoring (PRM). The quantitative confirmation of the 20 target proteins, and these included extraction of proteins, trypsin digestion, LC-MS/MS analysis and secondary MS data was then conducted. All the data were retrieved by Maxquant (v1.6.15.0) and these were processed using the Skyline 21.2 software package. PRM employs the peak areas in order to quantify the protein data obtained. By using differential analysis, when *P* < 0.05, the differential protein expression levels which exceeded 1.5 was taken as the significant upregulation change value. When it was less than 1, this was regarded as a significant downregulation change. When combining the key pathway proteins and some enzymes as well as the related proteins studied with respect to HF, this could be biased for screening the candidate proteins.

## Results

### Patient characteristics

The characteristics of the patient in this study are described in Table 1. No significant differences in the height, weight, age, Cr, UA, GFR, AST, ALT, HbA1c, Glu, WBC, Hb, PLT, CHO, TG, HDLC, LDLC, VLDLC, APOA1, APOB and Lpa between the MI-HF and MI-WHF groups were observed (*P* > 0.05).

**Table 1:** A comparison of the MI-HF and MI-WHF groups of patients (median).

	MI-HF group	MI-WHF group	*Z* value	*P-*value
N	10	10	—	—
High (cm)	165 (163, 167.75)	164 (160.5, 165)	63	0.34
Weight (kg)	64 (62.25, 66.5)	63.75 (61.5, 65)	57	0.622
Age (year)	60 (55.75, 75.5)	57.5 (53.25, 71.5)	59.5	0.496
Smokers	6	8	−	0.628
Drinkers	7	6	−	1.000
Hypertension	6	7	−	1.000
Diabetes	6	5	−	1.000
Cr (µmol/L)	80.5 (73, 97.25)	92 (76.5, 98)	43	0.622
UA (µmol/L)	362 (337.25, 419.75)	459.5 (360.75, 499)	41	0.529
GFR (%)	84.935 (69.285, 95.312)	78.78 (71.26, 91.64)	49	0.97
AST (U/L)	304.75 (72.725, 435.25)	204.55 (61, 303.75)	61	0.436
ALT (U/L)	71.15 (35.1, 105.5)	55 (35.6, 61)	61	0.436
HbA1c (%)	7 (5.9, 7.7)	5.8 (5.325, 6.675)	65.5	0.102
Glu (mmol/L)	6.59 (5.375, 7.992)	5.855 (5.69, 6.495)	53	0.853
WBC (×10^9^/L)	12.38 (11.043, 15.79)	12.245 (8.58, 13.093)	64	0.315
Hb (g/L)	127 (103.5, 150.5)	153 (137.25, 171.25)	23.5	0.049
PLT (×10^9^/L)	230.5 (196.5, 359.75)	246 (223.5, 263.5)	53	0.85
CHO (mmol/L)	5.01 (3.71, 5.805)	4.485 (3.373, 5.385)	57	0.631
TG (mmol/L)	1.6 (1.397, 2.49)	1.175 (0.892, 1.478)	73	0.089
HDLC (mmol/L)	0.95 (0.832, 1.228)	1.085 (0.923, 1.448)	36	0.315
LDLC (mmol/L)	3.015 (2.443, 3.318)	2.64 (2.24 2.968)	56.5	0.65
VLDLC (mmol/L)	0.73 (0.638, 1.135)	0.535 (0.408 0.67)	73	0.089
APOA1 (g/L)	1.27 (1.183, 1.36)	1.015 (0.942, 1.367)	65.5	0.256
APOB (g/L)	0.985 (0.792, 1.165)	0.875 (0.762, 0.995)	62.5	0.364
Lpa (nmol/L)	22.65 (19.65, 27.575)	49.95 (16.45, 201.025)	36	0.315

### Protein identification

**Figure 1:**
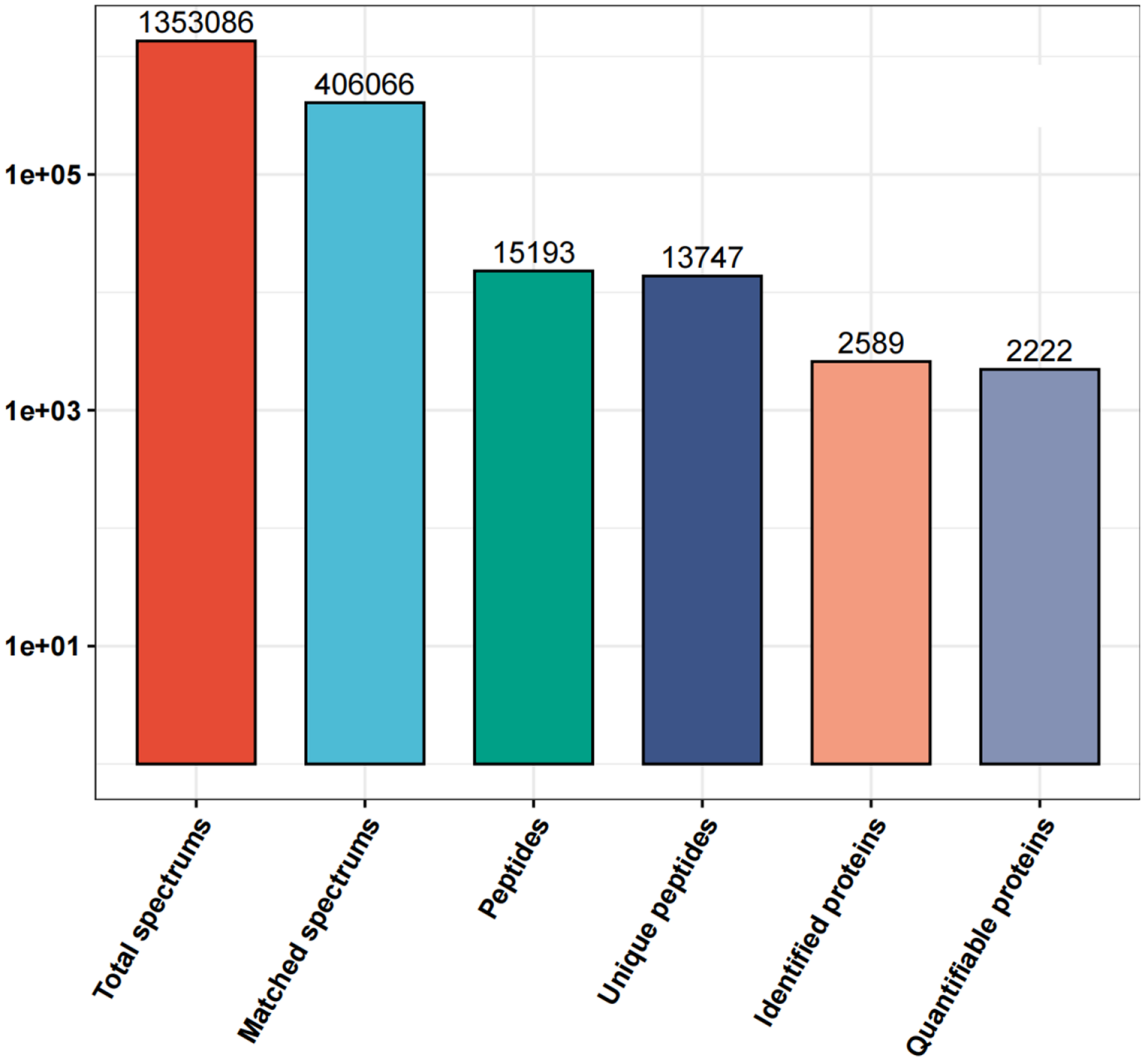
Details of the all the protein spectrums obtained from the proteins in the blood samples of the MI-WHF and MI-HF patients in this study. The matched spectrums refers to the number of effective spectrographs that matched the theoretical secondary spectrums obtained. The peptides refers to the number of identified peptides by matching the sequences. The unique peptides are the number of identified unique peptides after matching. The identified proteins refer to the number of identified proteins that were resolved and the quantifiable proteins are those that were quantified by using their specific peptides.

1,353,086 spectrums were obtained from the different proteins in the blood samples of the two groups of patients in this study. After analysis of the total protein data, the number of matched spectrums was reduced to 406,066. The utilization rate of spectrograms was 30.01% and 15,193 peptides were identified after analysis. 13,747 peptides with unique peptide segments were selected. In our study, 2,589 proteins were identified and of these 2,222 were quantifiable (Figure 1).

### Differential protein identification

**Figure 2:**
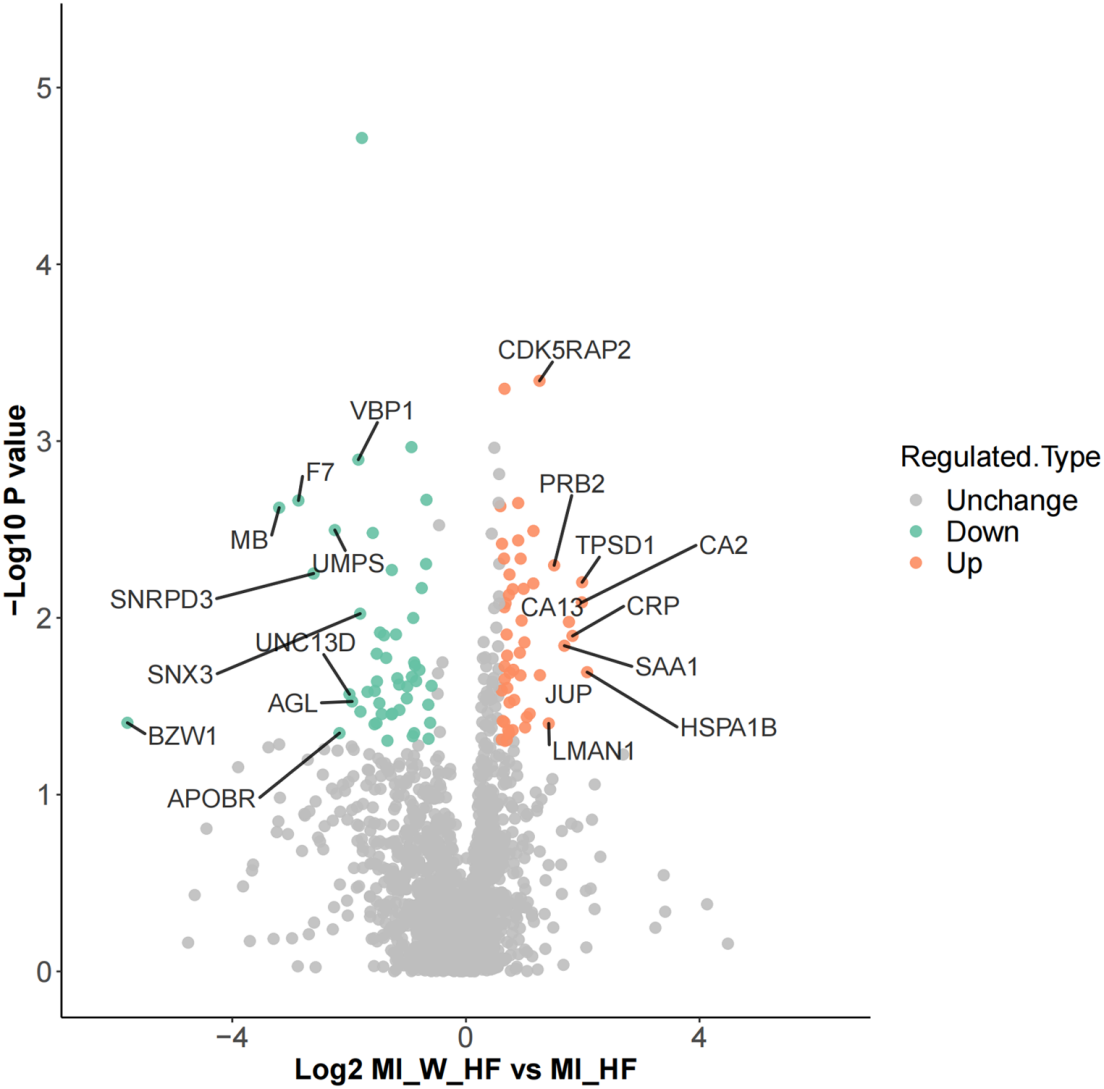
Differential protein identification. DEPs volcano showed there are 51 upregulated and 66 downregulated genes when the MI-HF and MI-WHF patient groups were compared. The green and red dots represent the downregulated and upregulated genes, respectively and the gray dots represent no change. The most differentially expressed proteins are clearly marked.

Differentially expressed proteins (DEPs) were identified and analyzed by their gene expression profiles and pathway enrichment, which were performed through three bioinformatic resources, namely GO, KEGG and protein domain analysis. A comparison of MI-HF and MI-W-HF patients found there were 51 and 66 upregulated and downregulated proteins, respectively (Figure 2 and Supplementary Table S1).

### Annotation and classification of the identified DEPs

**Figure 3:**
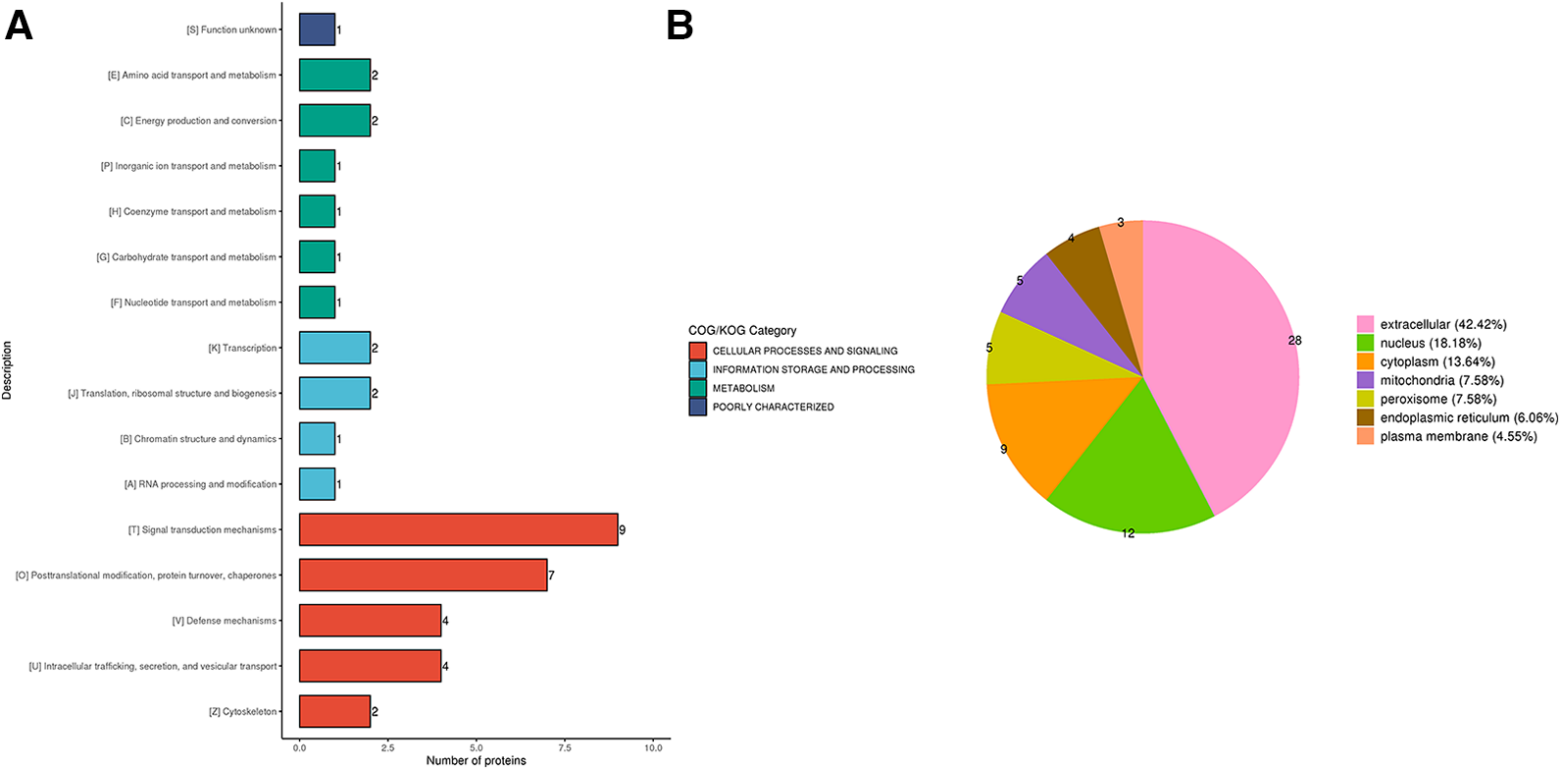
(**A**) the COG/KOG and (**B**) subcellular-based annotation and classification of the DEPs in terms of cellular process, information storage and processing, metabolism and poorly characterized proteins.

In order to annotate and classify the differential proteins function, COG/KOG categories were used to annotate their functional category distributions. The DEPs identified were classified into 4 COG/KOG categories. Cellular processes and signaling, information storage and processing and metabolism consisted of 47, 15 and 19 DEPs, respectively. One DEP was poorly characterized and no function was allocated to it (Figure 3A). Based on this, we annotated the proteins according to their subcellular structures using WoLF Psort software for eukaryotic cells and the PSORTb (v3.0) software for prokaryotic cells. The proteins were sorted as belonging to the extracellular, cytoplasm, nucleus, mitochondria, plasma membrane, cytoplasm/nucleus, endoplasmic reticulum and peroxisome with 32.48, 23.08, 17.95, 8.55, 5.13, 4.27, 4.27 and 4.27% of the total, respectively (Figure 3B).

### Enrichment analysis of DEPs

**Figure 4:**
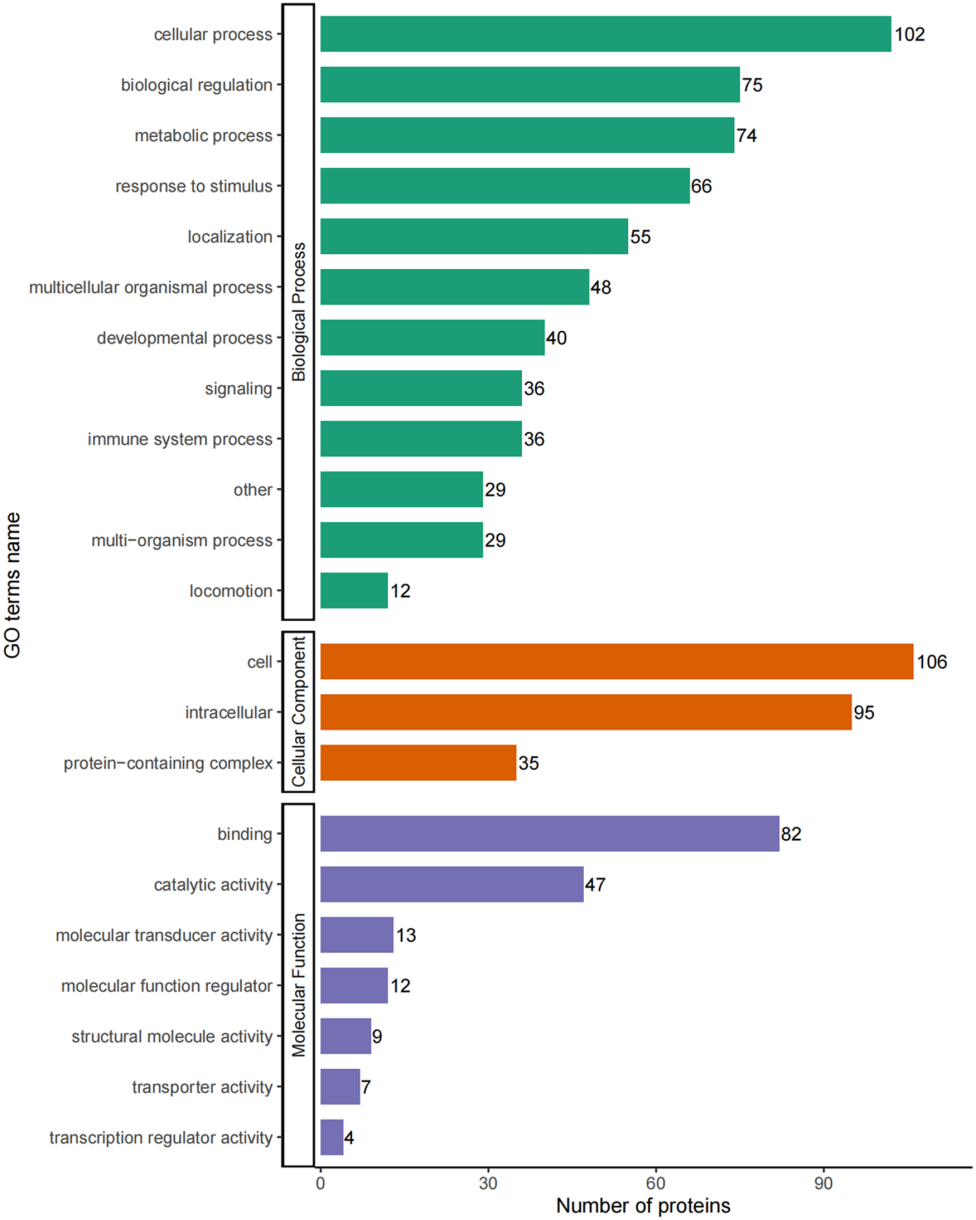
GO-based enrichment analysis of DEPs in terms of biological process, cellular component and molecular function.

The DEPs were classified by their GO secondary annotations by using database alignment in to three groups: biological processes (*n* = 602), cellular components (*n* = 236) and molecular functions (*n* = 174), which explained their biological roles. Among these, the most enriched GO terms were cellular process (102 proteins) in biological process, cell (106 proteins) in cellular component and binding (82 proteins) in molecular function (Figure 4).

### Functional enrichment analysis of DEPs

**Figure 5:**
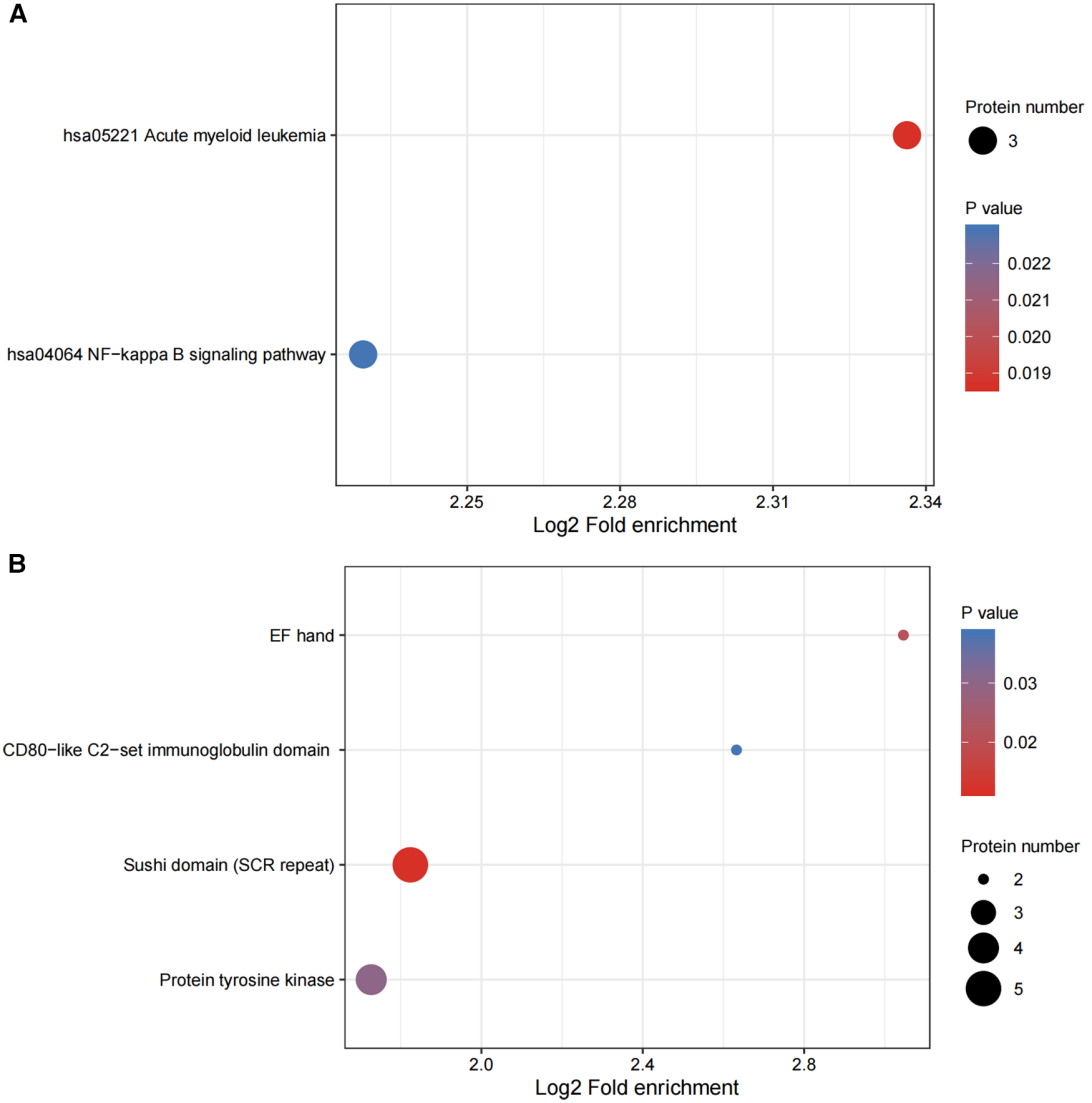
(**A,B**) functional enrichment of DEPs. Bubble plots were used to show the top 20 significantly enriched DEPs. The vertical axes are their functional classification or pathway and the horizontal axes are the Log2-transformed values for the proportion of DEPs in this functional type fold- enrichment over the proportion of the identified proteins. The color and size of the circles indicate the enrichment significance *P*-value and number of DEPs in their functional classes or pathways.

We conducted enrichment analysis of the KEGG pathway and protein domains by using Fisher's exact test. This was performed in order to finding whether the DEPs had significant enrichment trends in some of the functional types. The functional classification and pathways of the 20 most significantly enriched in DEPs were displayed in a bubble chart (Figures 5A,B).

### Cluster analysis

**Figure 6:**
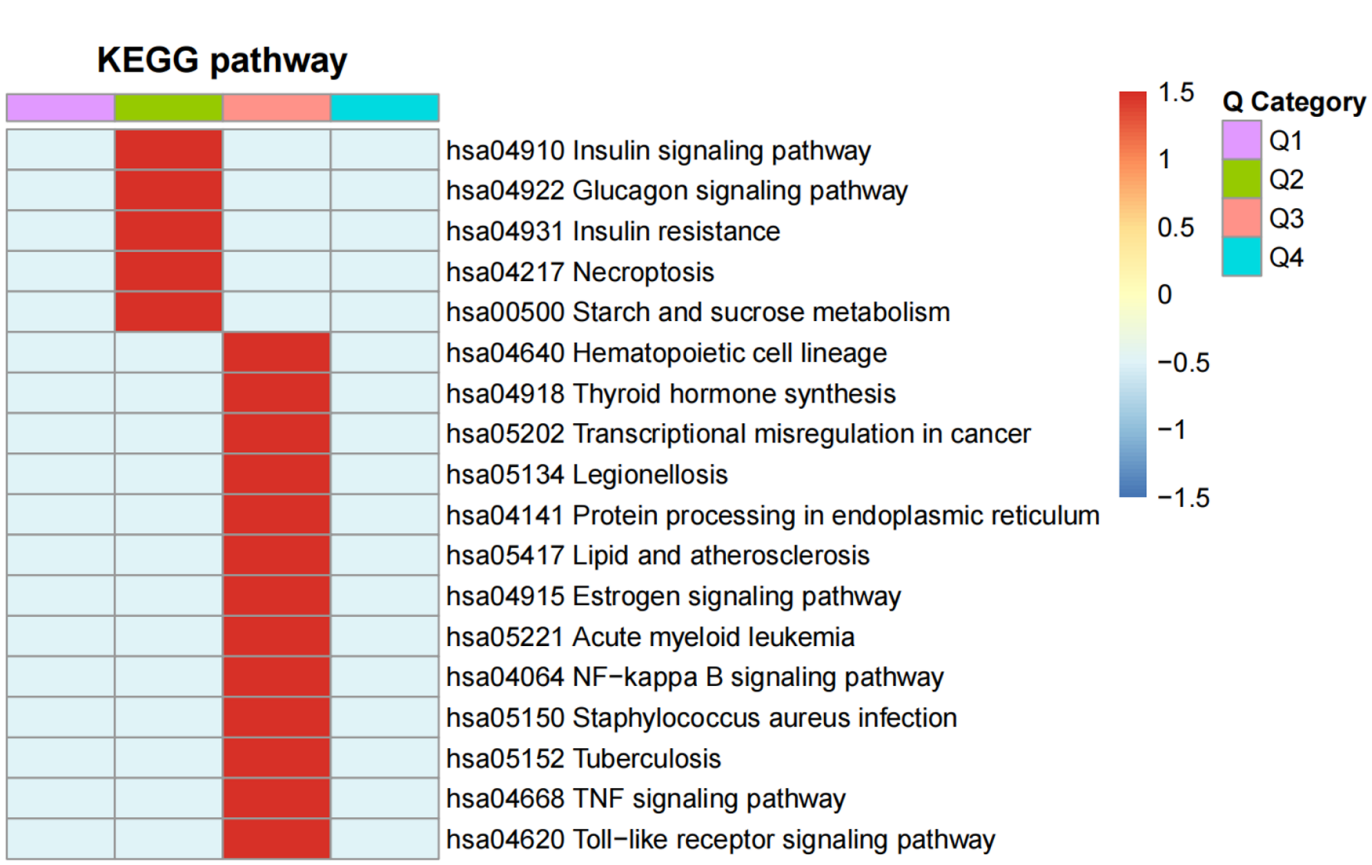
The functional enrichment clusters associated with the DEPS. A heatmap of the Fisher's exact test *P*-values from the enrichment analysis was drawn for the correlation functions in the MI-HF and MI-WHF patient groups by their fold-difference using hierarchical clustering. The Q1 and Q2 groups were the differential fold downregulated groups, where Q1 was the markedly enriched and Q2 was the generally enriched pathway downregulated. Groups Q3 and Q4 referred to the upregulation groups, respectively. The horizontal section of the heatmap is for the different fold groups, and the longitudinal section shows the description of the related functions of DEPs enriched these groups by the KEGG pathway analysis. Red and blue represent strong and weak enrichment of the different comparative DEPs groups, respectively.

The *P*-values from the enrichment analysis obtained using the Fisher's exact test were clustered for the correlation functions in different control groups by fold of difference using hierarchical clustering and drawing them into a heatmap (Figure 6).

### Protein interaction network analysis

**Figure 7:**
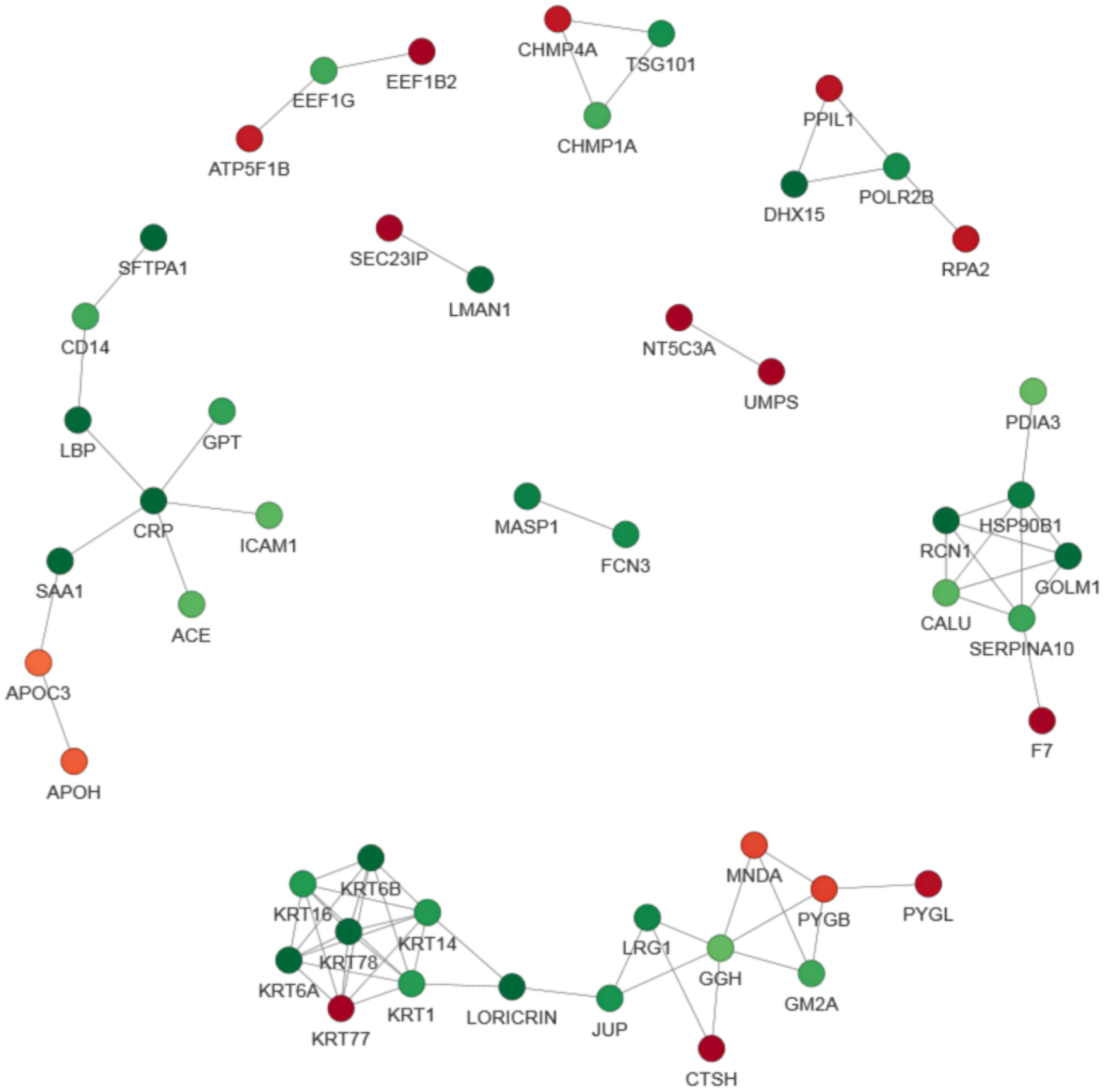
Protein–protein interaction (PPI) network analysis of DEPs. The STRING database was used to annotate the functional interactions of all the identified DEPs. The circles represent different proteins and the colors represent the differential expression of the proteins (green and red refer to downregulated and upregulated proteins, respectively), and the darker the color, the greater the difference. In order to clearly show the interaction relationship between proteins, we screened the top 49 closest interacting proteins in order to map the protein interaction network, presenting a global view of their diverse cellular functions. These DEPs were involved in acute myeloid leukemia and the NF−kappa B signaling pathway.

Either the differential protein database number or sequence of the protein were selected in the different groups by using the difference multiple, 1.5. Those with a confidence score >0.7 (high confidence) were extracted after comparing them with the STRING (v.11.0) protein interaction network database.The results were visualized by using the R package “network D3” tool. We screened of the top 49 closest interaction relationship proteins in order to map the protein interaction network and the DEPs involved in acute myeloid leukemia and the NF−kappa B signaling pathway were the most prominent (Figure 7).

### Heatmap results of PRM

During the experimental design, 2 unique peptides per protein were quantified but only one peptide was identified for sensitivity. However, 3 or more unique peptides were used to quantify each protein and PRM was used to quantify their peak areas. PRM quantification was performed for 20 proteins, but only 16 target proteins are presented and 14 proteins were found to have the same trend consistency by using the tandem mass tag technique. We found that the expression trend of each protein in the different groups was basically consistent with that obtained using their thermograms (Table 2).

**Table 2:** The verification results of the target proteins.

Protein accession no.	Protein description	Gene name	MI-HF/MI_WHF *P*-value	MI-HF/MI_WHF Ratio PRO	MI-HF/MI_WHF Trend consistency
A0A5F9ZHM4	L-lactate dehydrogenase OS = Homo sapiens OX = 9,606 GN = LDHB PE = 1 SV = 1	LDHB	2.28e−02	0.99	No
B0QYF8	Myoglobin (Fragment) OS = Homo sapiens OX = 9,606 GN = MB PE = 1 SV = 1	MB	6.73 × 10^−3^	8.4	Yes
O60610	Protein diaphanous homolog 1 OS = Homo sapiens OX = 9,606 GN = DIAPH1 PE = 1 SV = 2	DIAPH1	3.30 × 10^−1^	1.11	Yes
O95497	Pantetheinase OS = Homo sapiens OX = 9,606 GN = VNN1 PE = 1 SV = 2	VNN1	9.42 × 10^−1^	2.3	Yes
P00505	Aspartate aminotransferase, mitochondrial OS = Homo sapiens OX = 9,606 GN = GOT2 PE = 1 SV = 3	GOT2	2.27 × 10^−3^	5.14	Yes
P02730	Band 3 anion transport protein OS = Homo sapiens OX = 9,606 GN = SLC4A1 PE = 1 SV = 3	SLC4A1	6.40 × 10^−2^	2.48	Yes
P02741	C-reactive protein OS = Homo sapiens OX = 9,606 GN = CRP PE = 1 SV = 1	CRP	3.35 × 10^−1^	0.28	Yes
P06732	Creatine kinase M-type OS = Homo sapiens OX = 9,606 GN = CKM PE = 1 SV = 2	CKM	4.38 × 10^−2^	2.99	Yes
P08294	Extracellular superoxide dismutase [Cu-Zn] OS = Homo sapiens OX = 9,606 GN = SOD3 PE = 1 SV = 2	SOD3	4.14 × 10^−1^	1.65	Yes
P08709	Coagulation factor VII OS = Homo sapiens OX = 9,606 GN = F7 PE = 1 SV = 1	F7	2.40 × 10^−1^	6.39	Yes
P09622	Dihydrolipoyl dehydrogenase, mitochondrial OS = Homo sapiens OX = 9,606 GN = DLD PE = 1 SV = 2	DLD	1.50 × 10^−1^	1.11	Yes
P15259	Phosphoglycerate mutase 2 OS = Homo sapiens OX = 9,606 GN = PGAM2 PE = 1 SV = 3	PGAM2	1.73 × 10^−1^	3.34	Yes
P17174	Aspartate aminotransferase, cytoplasmic OS = Homo sapiens OX = 9,606 GN = GOT1 PE = 1 SV = 3	GOT1	2.74 × 10^−1^	1.74	Yes
P41226	Ubiquitin-like modifier-activating enzyme 7 OS = Homo sapiens OX = 9,606 GN = UBA7 PE = 1 SV = 2	UBA7	2.38 × 10^−1^	1.82	Yes
P48506	Glutamate–cysteine ligase catalytic subunit OS = Homo sapiens OX = 9,606 GN = GCLC PE = 1 SV = 2	GCLC	4.19 × 10^−1^	0.38	No
Q9Y4L1	Hypoxia up-regulated protein 1 OS = Homo sapiens OX = 9,606 GN = HYOU1 PE = 1 SV = 1	HYOU1	4.22 × 10^−2^	1.16	Yes

### Power calculation

The numbers of cases analyzed in each group were not high. Therefore, we performed a power calculation to determine the minimum number of required biological variants in each group and provide power curves exhibiting the minimum % effect size measurable as a function of sample size with at least 80% power at *P* < 0.05 and *P* < 0.01 levels of statistical significance (Supplementary Figures S1A,B). These were performed in R language using the pwr R software package.

## Discussion

AMI is a life-threatening condition that leads to morbidity and mortality, especially in the older generation (20). The incidence of HF in China is projected to rise over the coming years, as the prevalence of risk factors, such as AMI, rises (21). Unfortunately, there is still no precise definition of the etiology and mechanism of AMI-associated HF (22, 23). Consequently, exploring potential markers to determine HF after AMI has become essential to guarantee that high-quality cardiac treatment can be offered to patients who suffer from this condition.

In this study, we performed a comprehensive proteomic profiling and PRM analysis of plasma obtained from patients with MI-WHF and MI-HF. A total of 2,589 proteins were identified and 2,222 proteins were quantified in both MI-WHF and MI-HF groups by using the plasma proteome. 117 DEPs (≥1.5-fold, *P *< 0.05) were further explored and compared between the two groups. The results suggested that a wide range of proteins might be associated to several pathophysiological processes during AMI with HF. PRM analysis of 16 proteins was performed to further verify the expression levels of the target proteins. As a result, 14 proteins (MB, DIAPH1, VNN1, GOT2, SLC4A1, CRP, CKM, SOD3, F7, DLD, PGAM2, GOT1, UBA7 and HYOU1) were found to be involved during the process and presentation of MI-HF. A study by Hu et al. showed that VNN1 may be a promising therapeutic candidate against atherosclerosis (24). Another study showed that PGAM2 may be a promising new biomarker for evaluation of the severity of HF and it may be useful to judge the severity during differential diagnosis of this condition (25). In addition, constitutive overexpression of PGAM2 was shown to modify energy metabolism and reduced stress resistance on mice hearts (26). A cross-sectional study for 1,047 patients indicated that extracellular superoxide dismutase (Ec-SOD) might be a potential link between left ventricular structure remodeling and the development of subsequent HF in patients with cardiovascular disease (27). A loss of function mutation of SOD3 in rats induced chronic renal failure that develops during the first 5 months of age and this is accompanied by severe systemic hypertension and moderate LV hypertrophy (28). However, there are no studies on the relationship between the above PRM-associated proteins and HF after MI.

AMI is still one of the top causes of death worldwide (28). The fatal causes of AMI include malignant arrhythmias, acute HF and heart rupture with the first two being the most common. However, not all MI patients will lead to HF, and there may be a relationship with the site and area of infarction as well as the presence of inflammatory and other factors. However, with a similar site of infarction, there are still some patients who are prone to HF, and the reasons and mechanisms of this remains unclear.

In order to analyze the gene profiles of AMI patients, Xue et al. (29) performed a gene set enrichment analysis (GSEA), and obtained “TNFA_SIGNALING_VIA_NFKB” (enrichment score = 0.57), and they were able to experimentally validate their results. Briefly, “TNFA_SIGNALING_VIA_NFKB” are the genes regulated by nuclear factor kappa B (NF-kB) in response to TNF, NFkB and other inflammatory cytokines (30). These had all been previously shown to be enhanced in AMI patients (31). The AUC of the serum levels of TNF-α for predicting the occurrence of acute ST-elevated MI was 0.852 (32), and a TNF-α inhibitor was able to reduce the infarct area (33).

The NF-κB signaling pathway refers to the NF-κB family of mammalian transcription factors in mammals, consisting of P50 (treatment product of P105, both referred to as NF-κB 1), P52 (treatment product of p100, both called NF-κB 2), REL (alternatively called cREL), Rel-A (or P65) and Rel-B (34). These proteins dimerize and form a functional NF-κB, which is found in almost all animal cell types. It is involved in many cellular processes and is synthesized in response to stimuli such as stress, cytokines, free radicals, heavy metals, UV irradiation, oxidized LDL and bacterial and viral antigens (34). NF-κB is central to the immune response to infection (35). Incorrect regulation of NF-κB is also associated with cancer (36), inflammation (37), autoimmune (38) diseases, septic shock (39), viral infection (40), differentiation (41), apoptosis, ferroptosis (42) and cardiovascular diseases (43). NF-κB has been shown to be involved in the process of HF after ischaemia/re-perfusion (44). Emerging evidence shows that ferroptosis occurs during AMI (45) and HF (46). In this study, SLC4A1 and NF-κB were found to be enriched in AMI patients with HF (Figure 5A and Table 2). Lnc- SLC4A1 −1 functions in regulating the NF-κB pathway to mediate the occurrence of diseases such as cancer (47). NF-κB is an inducible transcription factor and it plays a key role in regulating the development and homeostasis of the immune system and in coordinating the inflammatory response (48) which is one of the main mechanisms of AMI and HF (49).

There are some limitations associated with this study. Firstly, this was only a cross-sectional study. Secondly, being a human study, several factors were not able to be controlled and it would need to be validated with cells *in vitro* as well as the use of animal models. Thirdly, although a large number of proteins were detected, only a small percentage of these were quantified and some key ones may have been overlooked. Future studies will be performed to address these limitations.

In conclusion, DEPs in the plasma of MI-HF patients and MI-WHF patients were quantitatively assessed and key proteins involved in the NF-κB signaling pathway as well as acute myeloid leukocytes were shown to be elevated in AMI with HF. PRM of the DEPs revealed that the expression trend of each protein between MI-HF and MI-W-HF was consistent with that of the protein thermogram obtained. These proteins may serve as candidate markers for patients AMI with HF as well as serving as potential novel control targets to elucidate the pathophysiology of this condition.

## Data Availability

The raw data supporting the conclusions of this article will be made available by the authors, without undue reservation.
